# A new, simple, highly scalable, and efficient protocol for genomic DNA extraction from diverse plant taxa

**DOI:** 10.1002/aps3.11413

**Published:** 2021-04-07

**Authors:** Evgeny V. Mavrodiev, Christopher Dervinis, William Mark Whitten, Matthew A. Gitzendanner, Matias Kirst, Sangtae Kim, Taliesin J. Kinser, Pamela S. Soltis, Douglas E. Soltis

**Affiliations:** ^1^ Florida Museum of Natural History University of Florida Gainesville Florida 32611 USA; ^2^ School of Forest Resources and Conservation University of Florida Gainesville Florida 32611 USA; ^3^ Department of Biology University of Florida Gainesville Florida 32611 USA; ^4^ School of Biological Sciences and Chemistry Sungshin Women’s University 249‐1, Dongsun‐Dong 3‐Ga, Sungbuk‐Gu Seoul 136‐742 South Korea

**Keywords:** cetyltrimethylammonium bromide (CTAB), extraction, genomic DNA, next‐generation sequencing, plant

## Abstract

**Premise:**

Commonly used molecular techniques such as next‐generation sequencing require reliable methods to extract DNA quickly and efficiently. Secondary compounds within plant tissues make this requirement all the more challenging, often forcing researchers to test different extraction methods tailored to their particular species of interest in order to obtain large amounts of high‐quality genomic DNA. The opportunities provided by high‐throughput, next‐generation sequencing only exacerbate these problems, especially when trying to extract DNA from multiple species at the same time. Several methods have attempted to resolve the challenges of obtaining suitable DNA from plants; however, a rapid, high‐yield, high‐quality, and highly scalable DNA extraction method is still needed.

**Methods and Results:**

We present a rapid DNA extraction protocol that utilizes a buffer with relatively large amounts of cetyltrimethylammonium bromide (CTAB) and sodium chloride, combined with a silica maxi‐column cleanup of the extracted DNA. The new method is easy to implement using standard equipment and inexpensive reagents. The entire procedure (from grinding to the final elution) can be completed in less than two hours for a single sample and can be easily scaled to meet desired research goals. It works on diverse green plants with highly varied secondary chemistries (e.g., ferns, gymnosperms, and phylogenetically divergent angiosperms).

**Conclusions:**

Application of the protocol to various plant species yielded DNA of high quality in less than two hours and can be adjusted to extract DNA at large (maxi‐preps) or small (96‐well minipreps) scales. We anticipate that our method will be of wide utility for rapidly isolating large quantities of quality genomic DNA from diverse plant species and will have broad applications in phylogenetic studies utilizing PCR and short‐read DNA sequencing.

Molecular techniques such as PCR and next‐generation sequencing have become commonplace in botanical research, and these tools have expanded our understanding of many phenomena related to genome structure, gene function, and phylogenetic relationships (e.g., Michael and Jackson, 2013; An et al., 2019). Due to cellular structure and unique chemistries, extracting large quantities of high‐quality DNA from plants can be challenging. Several methods have attempted to resolve these challenges, but often these methods rely on long incubation times both during initial tissue lysis and later for alcohol precipitation of the DNA (e.g., Doyle and Doyle, 1987; Agbagwa et al., 2012), adding considerable time to the process. Even once sufficient DNA is obtained, further removal of contaminating compounds is sometimes needed (e.g., Fang et al., 1992; Vaillancourt and Buell, 2019). The need to extract DNA from many different species at the same time can exacerbate this problem. A rapid, simple, scalable, high‐yield DNA extraction method, broadly applicable across diverse plant taxa, is still needed.

The major goal of this study is the development of a rapid and simple extraction method capable of yielding large amounts of high‐quality genomic DNA that is suitable for use with common laboratory techniques such as PCR and short‐read sequencing (e.g., Illumina or BGI‐Seq). This new method is easy to implement using standard equipment and inexpensive reagents, and we show that it works well across a diverse array of plant taxa (e.g., the fern *Angiopteris*, the gymnosperm *Pinus*, and diverse flowering plants; Tables 1, 2; Appendix 1). In addition, the entire procedure from grinding to the final elution is fast and can be completed in less than two hours; it can also be easily scaled to obtain the desired amount of DNA or number of extractions needed for diverse downstream applications.

**TABLE 1:** List of species included in the initial testing of the described DNA extraction method, their current phylogenetic placements, total yield (TY) of DNA, and A260/280 ratio.

Species	Clade(s)‐Order/Family	TY (μg)	A260/280
*Agapanthus africanus*	Angiosperms‐Monocots‐Asparagales/Amaryllidaceae	240	1.62
*Angiopteris evecta*	Polypodiopsida‐Marattiales/Marattiaceae	137	1.58
*Anthurium podophyllum*	Angiosperms‐Monocots‐Alismatales/Araceae	160	1.70
*Aristolochia arborea*	Angiosperms‐Magnoliids‐Piperales/Aristolochiaceae	69	2.0
*Austrobaileya scandens*	Angiosperms‐ANA grade^a^‐Austrobaileyales/Austrobaileyaceae	36	1.6
*Bocconia frutescens*	Angiosperms‐Eudicots‐Ranunculales/Papaveraceae	37	1.5
*Bulnesia arborea*	Angiosperms‐Eudicots‐Zygophyllales/Zygophyllaceae	228	1.76
*Cananga odorata*	Angiosperms‐Magnoliids‐Magnoliales/Annonaceae	106	1.71
*Canella winterana*	Angiosperms‐Magnoliids‐Canellales/Canellaceae	298	1.7
*Ceratophyllum demersum*	Angiosperms‐Ceratophyllales/Ceratophyllaceae	154	1.71
*Chloranthus spicatus*	Angiosperms‐Chloranthales/Chloranthaceae	37	1.78
*Grevillea robusta*	Angiosperms‐Eudicots‐Proteales/Proteaceae	21	1.61
*Medicago lupulina*	Angiosperms‐Eudicots‐Fabales/Fabaceae	300	1.64
*Myrothamnus flabellifolia*	Angiosperms‐Eudicots‐Gunnerales/Myrothamnaceae	54	1.19
*Pinus taeda*	Gymnosperms‐Pinales/Pinaceae	242	1.81
*Piper nigrum*	Angiosperms‐Magnoliids‐Piperales/Piperaceae	53	1.73
*Pisum sativum*	Angiosperms‐Eudicots‐Fabales/Fabaceae	21	1.65
*Tragopogon pratensis*	Angiosperms‐Eudicots‐Asterales/Asteraceae	50	1.54
*Triticum aestivum*	Angiosperms‐Monocots‐Poales/Poaceae	125	1.4
*Ximenia americana*	Angiosperms‐Eudicots‐Santalales/Olacaceae	70	1.89

^a^ Note the ANA grade is referred to rather than a clade.

**TABLE 2:** List of species from which DNA extractions were obtained and used for short‐read BGISEQ‐500 sequencing, with their current phylogenetic placements.^a^

Species	Clade‐Order/Family	Raw reads	Reads passing filter^b^	% Reads passing filter^b^
*Angiopteris evecta*	Pteridophyta‐Marattiales/Marattiaceae	53,808,547,800	49,475,484,234	92
*Aristolochia arborea*	Magnoliids‐Piperales/Aristolochiaceae	61,054,941,000	54,854,809,020	90
*Asarum* sp.	Magnoliids‐Piperales/Aristolochiaceae	61,998,886,800	56,063,573,874	90
*Austrobaileya scandens*	ANA grade^c^‐Austrobaileyales/Austrobaileyaceae	59,341,154,200	54,956,693,484	93
*Bocconia frutescens*	Eudicots‐Ranunculales/Papaveraceae	69,179,975,000	60,096,338,280	87
*Cananga odorata*	Magnoliids‐Magnoliales/Annonaceae	57,835,832,400	52,518,276,756	91
*Canella winterana*	Magnoliids‐Canellales/Canellaceae	53,768,184,000	49,775,646,294	93
*Ceratophyllum demersum*	Ceratophyllales/Ceratophyllaceae	41,027,080,200	35,637,265,620	87
*Chloranthus spicatus*	Chloranthales/Chloranthaceae	65,229,023,800	59,789,551,140	92
*Cinnamomum camphora*	Magnoliids‐Laurales/Lauraceae	86,059,114,800	74,104,070,832	86
*Grevillea robusta*	Eudicots‐Proteales/Proteaceae	65,297,652,400	59,348,285,766	91
*Illicium parviflorum*	ANA grade^c^‐Austrobaileyales/Schisandraceae	57,874,565,800	49,862,457,612	76
*Myrothamnus flabellifolia*	Eudicots‐Gunnerales/Myrothamnaceae	60,268,483,200	53,742,182,274	89
*Piper nigrum*	Magnoliids‐Piperales/Piperaceae	52,821,680,800	47,991,427,902	91
*Platanus occidentalis*	Eudicots‐Proteales/Platanaceae	66,404,073,400	42,133,608,192	63
*Rollinia mucosa*	Magnoliids‐Magnoliales/Annonaceae	60,770,396,800	54,384,201,234	89
*Santalum album*	Eudicots‐Santalales/Santalaceae	73,949,103,600	39,089,009,088	53
*Thalictrum pubescens*	Eudicots‐Ranunculales/Ranunculaceae	68,683,202,600	63,586,651,392	93
*Thalictrum* sp.	Eudicots‐Ranunculales/Ranunculaceae	131,902,533,800	122,655,347,694	93
*Ximenia americana*	Eudicots‐Zygophyllales/Zygophyllaceae	64,917,979,200	55,911,261,978	86

^a^ All species were sequenced in one‐half lane on a BGISEQ‐500 instrument, except *Thalictrum* sp., where a full lane was used.

^b^ The number of reads passing Q30.

^c^ Note the ANA grade is referred to rather than a clade.

## METHODS AND RESULTS

### Extraction protocol

Reagents, recipes, and a stepwise protocol can be found in Appendices 2 and 3. In short, the entire procedure is a re‐scaling of a modified cetyltrimethylammonium bromide (CTAB) method of DNA extraction (Doyle and Doyle, 1987; Agbagwa et al., 2012) utilizing a buffer with relatively large amounts of CTAB and sodium chloride (originally described in Agbagwa et al., 2012; hereafter referred to as “CTAB buffer”). However, our protocol offers a significantly decreased extraction time because (1) it does not require long incubations during lysis and (2) it uses silica‐membrane columns without alcohol precipitation of the DNA. Here, we describe the protocol for use with EconoSpin All‐In‐One Silica Maxi Spin Columns (catalog no. 2040‐050; Epoch Life Sciences, Missouri City, Texas, USA), but the method can be easily scaled for use with the Mini (Epoch 1910‐050/250) or Midi (Epoch 2030‐050) Spin Columns by adjusting the amount of starting material and extraction buffer. The extraction could even be performed in a 96‐well format through use of deep‐well plates and the 96‐well filter plate (Epoch 2020‐001).

Due to the high concentrations of salt and CTAB in the extraction buffer (3% CTAB, 4 M NaCl, 20 mM EDTA, and 100 mM Tris [pH 8]), preparation of the buffer requires incubation overnight at 60–70°C to dissolve the components.

#### Tissue disruption

Place up to 4 g of leaf tissue (preferably young, developing leaves) and 0.5 g of sand into a mortar along with 10 mL of CTAB buffer and 100 µL of proteinase K (20 mg/mL). Depending on the amount and nature of the tissue (e.g., coriaceous, soft), as well as the species under investigation, the amount of buffer can be adjusted to facilitate efficient grinding. Grind the tissue with a pestle until homogeneous and transfer the slurry into a chloroform‐resistant 50‐mL centrifuge tube. Bring the solution up to a volume of 25 mL by adding more CTAB buffer and then add 100 µL of 2‐mercaptoethanol (0.4% final concentration) and mix gently. Proceed directly to the chloroform extraction step without incubation as incubation in this formulation of the CTAB buffer will lead to DNA damage, resulting in the loss of yield and DNA integrity.

#### DNA extraction and isolation

To remove organic compounds such as proteins, extract the lysate by adding 25 mL of chloroform/isoamyl alcohol (24 : 1 v/v) to the 50‐mL tube, cap the tube, and gently mix by inverting 10 times every 1–3 min for at least 10 min. Centrifuge the tube for 3 min at ca. 2000 × *g* (3000 rpm on a Centra‐GP8 centrifuge; Thermo Fisher Scientific, Waltham, Massachusetts, USA) to separate the phases. Transfer the aqueous phase (top layer, usually 20 mL) to a new 50‐mL tube using a large‐bore pipette. At this point, the DNA is further cleaned by binding the DNA to a silica column. To facilitate binding, the aqueous phase is mixed with an equal volume of binding buffer (5 M guanidine hydrochloride, 30% isopropanol, or commercial equivalent [Qiagen Buffer PB; QIAGEN, Germantown, Maryland, USA]). Because the binding of DNA to silica membranes is pH‐dependent, add a sufficient quantity (usually 600 µL) of 3 M sodium acetate (pH 5.2) to the CTAB/binding buffer mix to bring the pH to approximately 5.5, using pH test strips to measure the pH (e.g., Fisher #1008576, Thermo Fisher Scientific). Failure to adjust the pH will result in loss of yield.

#### DNA purification, elution, and storage

Insert two EconoSpin All‐In‐One Silica Membrane Maxi Spin Columns (Epoch Life Sciences) into two new 50‐mL tubes and transfer the CTAB/binding buffer/3 M sodium acetate mixture evenly between the columns (ca. 20–25 mL to each column). Centrifuge for 3 min at 2000 × *g* and then remove the column, discard the flow‐through, and then place the column back into the same 50‐mL tube. Please note that the time of centrifugation will depend on the viscosity of the solution; some plant species yielding viscous solutions (e.g., *Illicium floridanum* J. Ellis) may require longer spins (up to 6 min).

To remove salts, clean the columns by adding 25 mL of a wash buffer (10 mM Tris‐HCl [pH 7.5], 80% EtOH, or a commercial equivalent [e.g., QIAGEN Buffer PE]) to each column and then centrifuge at 2000 × *g* for 3 min. Remove the column, discard the flow‐through, and replace the column into the same tube. Repeat this wash step after discarding the flow‐through.

Dry the columns by placing them into the empty 50‐mL tubes and centrifuging for 5 min at 2000 × *g* to remove any remaining wash buffer from the column before proceeding to the elution step. It is important that all traces of alcohol are removed or yield will be decreased. Once the column is dry, place the columns into new 50‐mL tubes and add 600 µL of pre‐warmed (70°C) Tris‐EDTA (TE) buffer. Incubate the tubes in a 70°C water bath (recommended) or oven for at least 20 min and then centrifuge at 2000 × *g* for 3 min to elute. Remove the eluate to a 1.7‐mL microfuge tube. A second elution may be performed but a new tube should be used, and it should be kept separate because the concentration can vary between elutions.

We also strongly recommend that the eluted DNA be treated with RNase A by adding 20 µL of the enzyme at 10 mg/mL and incubating for 20 min at room temperature with occasional gentle mixing.

The DNA is then quantified (using the Qubit DNA BR assay and NanoDrop One [Thermo Fisher Scientific]) and the size estimated (Agilent 2200 DNA TapeStation; Agilent Genomics, Santa Clara, California, USA) (see below); it can then be used for further molecular applications (e.g., PCR, sequencing) or stored at −80°C for long‐term storage.

### Species and quality assessments

To test the versatility of our method, DNA was isolated from a wide range of species representing ferns, gymnosperms, and angiosperms (Table 1). After extraction, the DNA was evaluated on an Agilent 2200 DNA TapeStation (Agilent Genomics) and the concentration measured with a Qubit DNA BR assay (Thermo Fisher Scientific) to determine the total yield in micrograms (Table 1). A NanoDrop One (Thermo Fisher Scientific) was used according to the manufacturer’s instructions to determine A260/280 ratios (Table 1).

**FIGURE 1:**
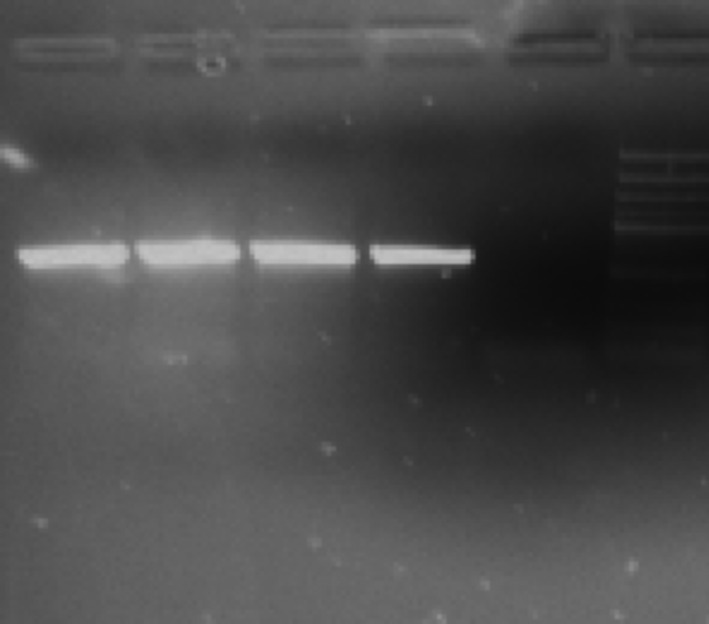
A 1.2% agarose gel showing the expected, 559‐bp PCR products using primers targeting the large subunit of ribulose‐1,5‐bisphosphate carboxylase‐oxygenase (*rbcL*) in DNA isolated from *Bulnesia arborea* (lane 1), *Medicago lupulina* (lane 2), *Anthurium podophyllum* (lane 3), and *Agapanthus africanus* (lane 4) (Table 1, Appendix 1). No template control is shown in lane 5, and DNA marker HyperLadder 100 bp (Bioline Reagents Ltd., London, United Kingdom) is shown in lane 6.

To assess the performance of our protocol, DNA was isolated from the angiosperms *Agapanthus africanus* (L.) Hoffmanns., *Anthurium podophyllum* (Schltdl. & Cham.) Kunth, *Bulnesia arborea* (Jacq.) Engl., and *Medicago lupulina* L. (Table 1, Appendix 1). One microliter of the eluted DNA was used as a template for PCR of the plastid gene encoding the large subunit of ribulose‐1,5‐bisphosphate carboxylase‐oxygenase (*rbcL*), using primers and conditions as described by Kress et al. (2009). All samples produced PCR products of the expected size of 559 bp (Fig. 1), indicating that DNA isolated using our method is free from PCR inhibitors.

DNA obtained by this protocol, from the species listed in Table 2, was successfully used to generate short‐read (whole‐genome sequencing) libraries using an MGIEasy DNA Library Preparation Kit (MGI Tech Co., Shenzhen, China) and sequenced (2 × 100 bp) on a BGISEQ‐500 by BGI Genomics (Shenzhen, Guangdong, China), which typically produces 120 Gbp/lane. All species in this study were sequenced in one‐half lane with the exception of the unidentified *Thalictrum* species, which was loaded into a full lane. The number of raw and filtered reads for these libraries can be found in Table 2. Libraries produced from DNA extracted with this protocol performed well during sequencing, producing the expected number of reads and with a Q30 quality filtration rate that ranged from 53–93% with a median of 90% (Table 2). Sequences from these libraries are currently undergoing assembly and annotation as part of a larger project at the University of Florida, the results of which will be released when ready for publication. Complete taxonomic names and voucher information for all species that were used in this study can be found in Appendix 1.

## CONCLUSIONS

The protocol described here produced large quantities of genomic DNA (for all plant species sampled). The quantity and quality of DNA were sufficient to generate both PCR‐based and whole‐genome short‐read sequences.

Our protocol implements attributes of the high‐salt CTAB buffer of established DNA extraction methods (e.g., Agbagwa et al., 2012); for example, the high concentration of NaCl successfully precipitates abundant polysaccharides (Fang et al., 1992), and 2‐mercaptoethanol binds to polyphenols, preventing them from binding to DNA (Mace et al., 2003).

Mark Whitten is credited with exploring the use of silica membrane spin columns, which are capable of binding large amounts of DNA, and suggesting the use of the high‐concentration CTAB and high‐NaCl buffer, which help lyse cell membranes. Combined, these features ensure large amounts of relatively clean DNA and avoid the need for precipitation, resulting in a quick and efficient protocol.

As the use of next‐generation sequencing becomes increasingly widespread, there is an increased need for methods that not only work well on challenging plant tissues, but also yield large amounts of high‐quality genomic DNA. This goal is all the more challenging in diverse plant species due to their complex secondary compounds, including tannins and polyphenolics, and because plant cells are more difficult than animal cells to break due to the presence of cell walls. Some protocols overcome these challenges via the use of liquid nitrogen, long incubation times, and/or alcohol to extract or precipitate the DNA (e.g., Agbagwa et al., 2012; Mayjonade et al., 2016). However, these steps can damage the DNA through mechanical fracturing, thus reducing the yield of high‐quality genomic DNA. By using high concentrations of NaCl and CTAB, our protocol simultaneously disrupts cells, protects the genomic DNA from mechanical damage, and prevents binding to phenolic compounds. Removing most organic compounds by the use of chloroform prior to binding of the DNA onto silica columns also removes most compounds that could potentially interfere with DNA binding; once the DNA is bound, washes of the silica columns remove any remaining contaminants.

Application of our protocol to diverse plant species yielded large amounts of high‐quality genomic DNA in less than two hours using inexpensive reagents. The protocol can be easily scaled to fit experimental needs, performs well across a variety of plant species, and can be set up in a 96‐well format. We are hopeful that our method will be of broad utility in diverse DNA sequencing applications, particularly with plant species and tissues considered difficult due to secondary compounds.

## Author Contribution

**Evgeny V. Mavrodiev:** Conceptualization (equal); Data curation (equal); Formal analysis (equal); Investigation (equal); Methodology (equal); Writing – original draft (equal); Writing – review & editing (equal). **Christopher Dervinis:** Conceptualization (equal); Data curation (equal); Writing – original draft (equal); Writing – review & editing (equal). **Matthew A. Gitzendanner:** Data curation (equal); Investigation (equal); Methodology (equal). **Matias Kirst:** Validation (equal); Writing – original draft (equal). **Sangtae Kim:** Data curation (equal); Formal analysis (equal); Methodology (equal). **Taliesin J. Kinser:** Data curation (equal); Formal analysis (equal); Resources (equal); Writing – original draft (equal); Writing – review & editing (equal). **Pamela S. Soltis:** Conceptualization (equal); Data curation (equal); Formal analysis (equal); Supervision (equal); Writing – original draft (equal); Writing – review & editing (equal). **Douglas E. Soltis:** Conceptualization (equal); Data curation (equal); Formal analysis (equal); Supervision (equal); Writing – original draft (equal); Writing – review & editing (equal). **William Mark Whitten:** Conceptualization (equal).

## APPENDIX 1 Complete taxonomic names and voucher information of all species involved in the study.

**Table:**

Species^a^	Herbarium: voucher no./barcode^b^
*Agapanthus africanus* (L.) Hoffmanns.	FLAS: Abbott 226858
*Angiopteris evecta* (G. Forst.) Hoffm.	FLAS: Whitten 5857
*Anthurium podophyllum* (Schltdl. & Cham.) Kunth	FLAS: Abbott 236011
*Aristolochia arborea* Linden	FLAS: Whitten 5851
*Asarum* L. sp.	FLAS: Whitten 5854
*Austrobaileya scandens* C. T. White	FLAS: Whitten 5849
*Bocconia frutescens* L.	FLAS: Whitten 5848
*Bulnesia arborea* (Jacq.) Engl.	FLAS: Moore 222665
*Cananga odorata* (Lam.) Hook. f. & Thomson	FLAS: Whitten 5850
*Canella winterana* (L.) Gaertn.	FLAS: Whitten 5853
*Ceratophyllum demersum* L.	FLAS: Whitten 5830
*Chloranthus spicatus* (Thunb.) Makino	FLAS: Whitten 5852
*Cinnamomum camphora* (L.) J. Presl	FLAS: Whitten 5843
*Grevillea robusta* A. Cunn. ex R. Br.	FLAS: Whitten 5844
*Illicium floridanum* J. Ellis	FLAS: Whitten 5833
*Illicium parviflorum* Michx.	FLAS: Whitten 5831
*Medicago lupulina* L.	FLAS: Abbott 214066
*Myrothamnus flabellifolius* Welw.	FLAS: Whitten 5845
*Pinus taeda* L.	FLAS: Abbot 8199
*Piper nigrum* L.	FLAS: Whitten 5855
*Pisum sativum* L.	FLAS: Lange 1403
*Platanus occidentalis* L.	FLAS: Whitten 5832
*Santalum album* L.	KEW: 2014‐204
*Thalictrum pubescens* Pursh	FLAS: Whitten 5367
*Thalictrum* sp.	FLAS: Whitten 5846
*Tragopogon pratensis* L.	Soltis lab.: Soltis, P. 3058‐4
*Triticum aestivum* L.	FLAS: Lange 1403
*Ximenia americana* L.	FLAS: Whitten 5834

^a^ Two samples are identified only to genus due to the high taxonomic complexity of the correspondent genera (*Asarum* [Aristolochiaceae, Piperales] and *Thalictrum* [Ranunculaceae, Ranunculales]).

^b^ Herbarium acronym or repository; herbarium acronyms are according to Index Herbariorum (Thiers, 2021).

## APPENDIX 2 Reagents and equipment for use with Mark Whitten’s protocol for genomic DNA extraction.

### List of reagents and equipment

Legume high‐salt CTAB buffer (see below for the recipe)

EconoSpin All‐In‐One Silica Membrane Maxi Spin Column (catalog no. 2040‐050; Epoch Life Sciences, Missouri City, Texas, USA)

Centrifuge Falcon tubes, 50 mL (any brand)

Binding buffer (Buffer PB) (see below)

Wash buffer (Buffer PE) (see below)

Tris‐EDTA buffer (TE), pH 8.0 (any brand)

3 M sodium acetate, pH 5.5 (any brand)

Chloroform/isoamyl acetate 24 : 1 (any brand)

Proteinase K (any brand)

2‐mercaptoethanol (any brand)

RNase A (20 mg/mL) (any brand)

Standard and disposable plastic transfer pipettes (any brand)

Water bath (any brand)

Microfuge tubes (1.7 µL) (any brand)

Qubit DNA BR assay (Thermo Fisher Scientific, Waltham, Massachusetts, USA)

Agilent 2200 DNA TapeStation (Agilent Genomics, Santa Clara, California, USA)

### Recipes and critical sources

**Legume high‐salt CTAB buffer** (Agbagwa et al., 2012):

For 25 mL:

3% CTAB = 0.75 g

4 M NaCl = 5.9 g

20 mM EDTA = 1 mL of 0.5 M stock

100 mM Tris pH 8.0 = 2.5 mL of 1 M stock

For 1 L:

3% CTAB = 30 g

4 M NaCl = 236 g

20 mM EDTA = 40 mL of 0.5 M stock

100 mM Tris = 100 mL of 1 M stock

NOTE: Tris buffer 1 M, pH 8.0 (Fisher BP1758‐500; Thermo Fisher Scientific); EDTA 0.5 M, pH 8.0 (Fisher BP2482‐500)

The buffer requires overnight incubation at 60–70°C for all of the CTAB and salt to dissolve. The buffer is very viscous, but the viscosity decreases after tissue grinding and incubation. Use disposable plastic transfer pipettes (e.g., Fisher 13‐711‐9AM) for pipetting.

**Binding buffer:**

For 25 mL:

5 M guanidine hydrochloride = 11.94 g

30% isopropanol = 7.5 mL of 100% stock

Alternately, you can use QIAGEN Buffer PB (catalog no. 19066; QIAGEN, Germantown, Maryland, USA).

**Wash buffer:**

For 25 mL:

10 mM Tris‐HCl, pH 7.5 = 250 µL of 1 M stock

80% EtOH = 20 mL of 100% stock

Alternately, you can use QIAGEN Wash Buffer PE (catalog no. 19065).

## APPENDIX 3 Mark Whitten’s protocol for genomic DNA extraction presented as an abbreviated list.

1. Place 4.0 g of fresh leaf tissue into a mortar. Add 10 mL of CTAB buffer, 0.5 g of sand, and 100 µL of proteinase K solution. Manually grind the tissues using a pestle.
2. Transfer the slurry to a 50‐mL centrifuge tube, add buffer for a total volume of 25 mL. Add 100 µL of 2‐mercaptoethanol to the same tube. Mix gently.
3. To each tube, add 25 mL of chloroform/isoamyl alcohol (24 : 1). Incubate tubes for 10 min at room temperature with occasional gentle mixing every 1–3 min.
4. Centrifuge for 3 min at 2000 × *g* to separate phases.
5. Using a large‐bore pipette, remove the aqueous (top) layer and transfer it to a new 50‐mL tube.
6. To the supernatant (should be ca. 20 mL), add an equal volume of binding buffer, plus 600 µL of 3 M sodium acetate (pH 5.5).
7. Cap the tube and mix gently.
8. Check the pH of the supernatant mix with test paper before proceeding. It should be approximately pH 5.5. Adjustment of the pH is critical for DNA binding to the silica column.
9. Prepare two 50‐mL tubes for silica column purification by inserting Maxi silica columns into each tube.
10. Fill both Maxi columns with the CTAB/binding buffer/sodium acetate mix. Centrifuge at 2000 × *g* for 3 min and discard the flow‐through.
11. Fill both columns with a wash buffer; centrifuge at 2000 × *g* for 3 min. Discard the flow‐through.
12. Repeat step 11.
13. Centrifuge the columns at 2000 × *g* for 5 min to dry columns and to remove all the wash buffer. It is very important to make sure the columns are dry before proceeding to elution.
14. Remove the columns and place each column in a new, clean 50‐mL tube. Add 600 µL of pre‐warmed low Tris‐EDTA buffer (TE), pH 8.0, to each column. Place tubes in a water bath (70°C) or warm oven for 20 min.
15. Centrifuge the tubes at 2000 × *g* for 3 min.
16. Remove the eluate to a 1.7‐µL microfuge tube. The eluted DNA solution will be relatively concentrated.
17. If desired, place the silica columns in new 50‐mL tubes and repeat elution. Keep the eluates separate.
18. Add 20 µL of the 20 mg/mL RNase A to each 1.7‐µL microfuge tube with the eluted DNA. Keep at room temperature for ca. 20 min and mix gently.
19. For ca. 24 h or less, keep the 1.7‐µL microfuge tubes with eluted DNA at 4°C.
20. Check the quality and quantity of the eluted DNA using a Qubit fluorometer and TapeStation.
21. Immediately use the extracted DNA or aliquot and store at −80°C.
